# A software tool for segmentation of the myocardium in CMR exams using level-set algorithm

**DOI:** 10.1186/1532-429X-18-S1-P113

**Published:** 2016-01-27

**Authors:** Azadeh Nazemoroaya, Abbas Nasiraei-Moghaddam

**Affiliations:** grid.411368.90000000406116995Amirkabir University of Technology, Tehran, Iran (the Islamic Republic of)

## Background

In many Cardiac MR Exams, we need to extract the myocardium area precisely, which is a labor-intensive task if performed manually. Furthermore, the manual segmentation is a source of error since it highly depends on the user experience. Several methods, therefore, have been introduced to facilitate and expedite this time-consuming procedure [1–3].We have implemented a software tool in processing of cardiac tagging images and calculation of strain, which is sensitive to segmentation especially in radial direction. Compared to other methods, such as active contours and snakes, "level-set" is usually less sensitive to the initialization and also can be splitted automatically and quickly to detect more than one connected component [4]. Hence, we used level-set as the core of our user-friendly software to make it robust to initialization and segmentation of MR images. The possibility of intensity inhomogeneity was also considered.

## Methods

The level-set framework has been exploited in order to do curve evolution to extract endocardium and epicardium. The Algorithm includes definition of an energy of the level-set functions and a bias field that accounts for the intensity inhomogeneity (ƒ (φ, c, b)). Segmentation is performed by minimizing the energy based on gradient flow equation, while updated c_i_ (distinct constants in disjoint regions of image Ω_i_) and b (bias field) are given as input parameters from previous iteration. In this implementation user acts as supervisor for the algorithm only for the first frame by applying some minor changes on the calculated contours. Thereafter borders of the myocardium in subsequent frames are calculated using the anatomical and dynamical information determined in the previous frame. To examine the accuracy and robustness of the software, the myocardium was extracted both manually and automatically in short axis view of 120 balanced SSFP MR images related to 4 series of data from healthy people.

## Results

**Figure 1:**
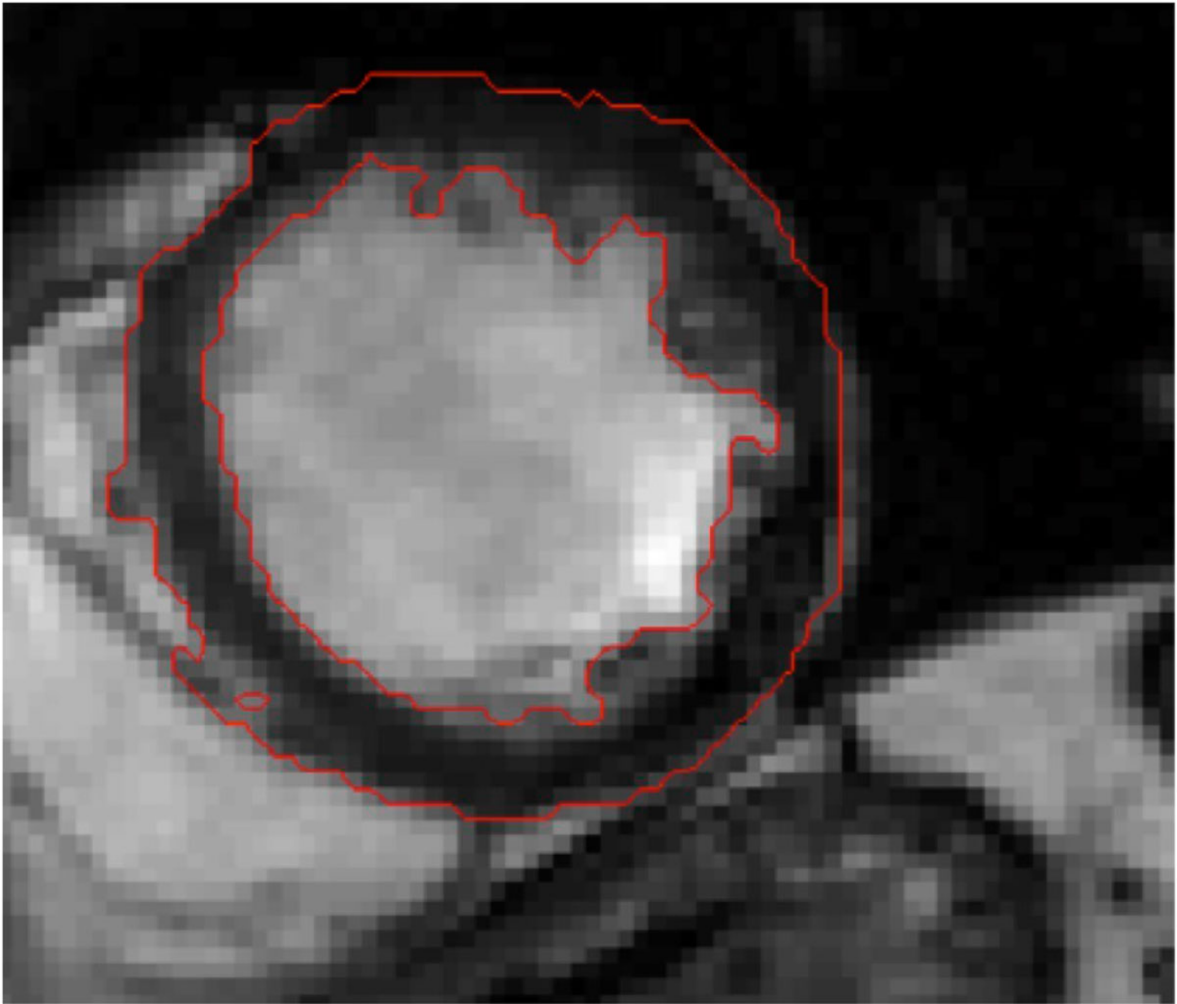
**A sample output of the software for the phase 1 out of 30**.

Figure 1 shows a sample output of the software**.** We evaluated the algorithm by calculating the mean sensitivity and the mean specificity, which were 0.918 and 0.959, respectively. The total time needed for extracting myocardium for each dataset was dramatically decreased by a factor of 7. The software coding was carried out entirely in MATLAB 2013a environment and the processor of used computer was 5th generation Intel^®^ Core™ i5 processors.

## Conclusions

A software tool for semi-automatic segmentation of the myocardium in MR images was developed, which needs no user interaction except for minor changes in the first frame. Validation has been performed on real data from healthy people. A study of segmentation errors by calculating the specificity and sensitivity shows satisfactory results. Also the time needed for extracting myocardium reduced dramatically.
